# Primary Renal Angiosarcoma with Extensive Necrosis: A Difficult Diagnosis

**DOI:** 10.1155/2014/416170

**Published:** 2014-07-15

**Authors:** Sohail Qayyum, Jignesh G. Parikh, Nadeem Zafar

**Affiliations:** Department of Pathology, University of Tennessee Health Science Center, Memphis, TN 38163, USA

## Abstract

Angiosarcoma of the kidney is an exceedingly rare and aggressive neoplasm. Very few cases have been reported in the English literature to date. We report a case of primary renal angiosarcoma with extensive necrosis and discuss its diagnostic difficulties. An 86-year-old male presented with a 12 cm necrotic renal mass and multiple pulmonary and hepatic nodules. A CT guided renal biopsy revealed extensive necrosis and few vascular channels lined by malignant endothelial cells. Diagnosis was given on a morphologic base and proven by an immunohistochemical study. Primary renal angiosarcoma should be included among the differential diagnosis of necrotic renal lesions.

## 1. Introduction

Angiosarcoma is a rare malignant tumor accounting for less than 2% of all soft tissue sarcomas [1]. Approximately one-third of primary angiosarcomas occur in skin, one-third in soft tissue, and the remaining one-third in other sites like bone, breast, and liver [2]. Primary renal angiosarcomas are very rare, while angiosarcomas involving the kidney usually represent metastasis from other sites [2–4]. It occurs most frequently between 60 and 70 years of age, with a male prediction [1, 4]. Here we report for the first time about a case of a primary renal angiosarcoma with extensive necrosis, which makes it a very challenging diagnosis.

## 2. Case Report

**Figure 1:**
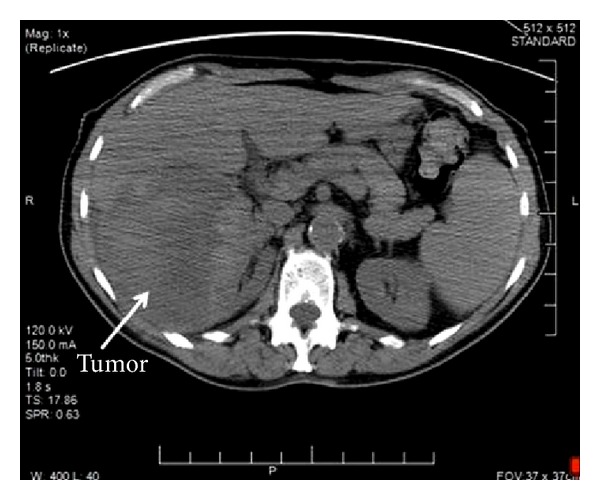


**Figure 2:**
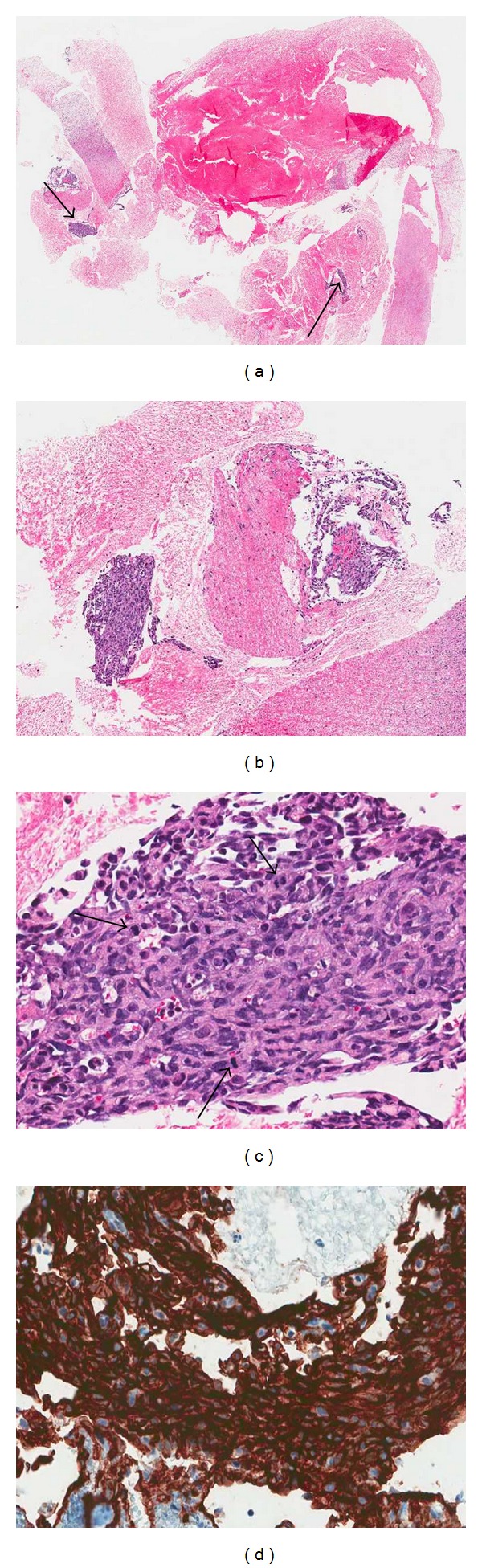
Microphotographs of a renal biopsy. (a) Scant tissue fragments with extensive necrosis (HE, ×40). (b) Scant tissue fragments with extensive necrosis (HE, ×100). (c) Multiple vascular channels lined by pleomorphic endothelial cells and increased mitotic figures (arrows) (HE, ×400). (d) Strong immunoreactivity for an endothelial marker CD34 (IHC, ×400).

An 86-year-old white/black male with a past medical history significant for stroke, hypertension, and squamous cell carcinoma of skin presented with the chief complaint of fatigue, dizziness, and generalized weakness and weight loss for the last few months. On examination his respiratory, gastrointestinal, cardiovascular, genitourinary, and central nervous systems were unremarkable. A chest X-ray showed multiple bilateral noncalcified lung nodules. Whole body CT scan revealed multiple hypervascular hepatic nodules and a right kidney mass measuring 12.3 × 9 × 8 cm (Figure 1), which appeared well encapsulated and partially necrotic, suspicious for primary renal cell carcinoma with pulmonary and hepatic metastasis. A CT guided renal biopsy was performed which revealed scant vascular fragments, which was nondiagnostic. Repeat biopsy revealed scant tissue fragments with extensive necrosis (Figures 2(a) and 2(b)) and multiple vascular channels lined by pleomorphic endothelial cells with increased mitotic figures (Figure 2(c)). Immunohistochemistry stains were positive for CD34 (Figure 2(d)) and CD31 affirming the vascular nature of this lesion and negative for AE1/3, CK8/18, CD10, and RCC. The diagnosis of primary angiosarcoma with extensive necrosis was entertained. Three surgical pathologists from our department were consulted on this case and concurred with the diagnosis. An outside consult to a well-known academic genitourinary pathology department was made and they too concurred with our diagnosis. Numerous bilateral pulmonary nodules and hepatic nodules were highly suspicious for metastatic angiosarcoma. Biopsies of these nodules were not possible as the patient denied further workup. He was seen by hematology/oncology and urology service, and a nephrectomy was recommended. However, the patient refused any aggressive intervention or surgery and, as per his decision, was given supportive treatment and referred for palliative care.

## 3. Discussion

Primary angiosarcoma of the kidney is an exceedingly rare malignant neoplasm [3, 4]. Our case has a unique feature of extensive necrosis and paucicellularity, which made it a very toilsome diagnosis. The patient had multiple pulmonary and hepatic nodules and a necrotic renal mass on imaging studies (Figure 1), which was initially thought to be a primary necrotic renal cell carcinoma with metastasis. Due to extensive necrosis, the first biopsy did not reveal sufficient material for diagnosis, while the second biopsy revealed only scarce tissue fragments containing the malignant lesion (Figures 2(a) and 2(b)). Due to the rarity of this tumor, intradepartmental and outside consultations were made to authenticate the diagnosis of primary angiosarcoma with extensive necrosis.

Angiosarcoma tumor cells are not well differentiated and endothelial cell markers are generally needed for diagnosis [1, 5]. The main differential diagnosis for necrotic primary renal angiosarcoma includes necrotic renal cell carcinoma [6]. Other necrotic conditions like metastatic carcinomas, tuberculosis, Rosai-Dorfman disease, malacoplakia, and xanthogranulomatous reaction to the staghorn calculus should also be excluded. In our case, tumor cells were positive for CD34 and CD31 (Figure 2(d)) and negative for AE1/3, CK8/18, CD10, and RCC. These immunohistochemical findings along with microscopic features helped us to rule out other possibilities and establish the diagnosis. Radiologic studies are needed to distinguish primary from metastatic lesions [1, 7]. The kidney is likely the primary origin of the angiosarcoma in our case, based on the single large lesion in kidney (Figure 1) and multiple metastatic nodules in liver and bilateral lungs on radiological studies.

Although risk factors for primary renal angiosarcoma have not been identified, angiosarcomas arising in other parts of the body have known predisposing factors. Exposure to arsenic, thorotrast, and polyvinyl chloride is associated with hepatic angiosarcomas, while radiation and posttreatment lymphedema are associated with soft tissue angiosarcomas [5, 8]. Our patient did not have a history of exposure to any of these risk factors. Similar to our case, most primary renal angiosarcomas have already metastasized at the time of diagnosis [1, 4, 9]. The most common symptoms at presentation are of hematuria, palpable mass, and flank pain [7, 9].

Due to the rarity of this lesion there are no standard therapies. Most of the reported cases have been treated with radical nephrectomy [1, 4]. The subsequent use of chemotherapy and/or radiotherapy is controversial, as some authors found adjuvant radiotherapy useful for local control, whereas others do not believe that they prolong survival [4]. Newer agents including recombinant interleukin-2, bevacizumab, and sorafenib have also been used recently [3]. Despite treatment the prognosis is usually very poor [4]. Even though it is recommended, our patient refused nephrectomy or aggressive treatment and chose to receive supportive care.

In summary, we present a rare case of primary renal angiosarcoma with extensive necrosis, which was initially thought to be a necrotic renal cell carcinoma clinically and radiologically. Due to the necrotic nature of this tumor, more than one biopsy was required and only few tumor cells were identified, which posed a great diagnostic challenge. Pathologist and radiologist must be aware of this rare aggressive malignancy while dealing with necrotic renal lesions.

## References

[1] Lopez B, Perez F, Alvarez JAC (2007). Renal primary angiosarcoma. *Clinical and Translational Oncology*.

[2] Lee CH, Park KU, Nah DY, Won KS (2003). Bilateral spontaneous pneumothorax during cytotoxic chemotherapy for
angiosarcoma of the scalp : a case report. *Journal of Korean Medical Science*.

[3] Yoshida K, Ito F, Nakazawa H (2009). A case of primary renal angiosarcoma. *Rare Tumors*.

[4] Leggio L, Addolorato G, Abenavoli L (2006). Primary renal angiosarcoma: a rare malignancy: a case report and review of the literature. *Urologic Oncology*.

[5] Johnson VV, Gaertner EM, Crothers BA (2002). Fine-needle aspiration of renal angiosarcoma. *Archives of Pathology and Laboratory Medicine*.

[6] Leibovitch I, Lev R, Mor Y, Golomb J, Dotan ZA, Ramon J (2001). Extensive necrosis in renal cell carcinoma specimens: potential clinical and prognostic implications. *Israel Medical Association Journal*.

[7] Brown JG, Folpe AL, Rao P (2010). Primary vascular tumors and tumor-like lesions of the kidney: a clinicopathologic analysis of 25 cases. *The American Journal of Surgical Pathology*.

[8] Hiratsuka Y, Nishimura H, Kajiwara I, Matsuoka H, Kawamura K (1997). Renal angiosarcoma: a case report. *International Journal of Urology*.

[9] Ribeiro de Souza OE, Etchebehere RM, Lima MA, Monti PR (2006). Primary renal angiosarcoma. *International Brazilian Journal of Urology*.

